# Insilico exploration *C*. *koseri* ATP synthase inhibitors by pharmacophore-based virtual screening, molecular docking and MD simulation

**DOI:** 10.1371/journal.pone.0308251

**Published:** 2024-08-22

**Authors:** Abdullah R. Alanzi, Alanazi A. Z., Khalid Alhazzani

**Affiliations:** 1 Department of Pharmacognosy, College of Pharmacy, King Saud University, Riyadh, Saudi Arabia; 2 Department of Pharmacology and Toxicology, College of Pharmacy, King Saud University, Riyadh, Saudi Arabia; Ahram Canadian University, EGYPT

## Abstract

*Citrobacter koseri* is a gram-negative rod that causes infections in people who have significant comorbidities and are immunocompromised. Antibiotic-resistant strains are becoming more common, which complicates infection treatment and highlights the need for innovative, effective drugs to fight these resistant strains. The enzyme complex ATP synthase participates in the adenosine triphosphate (ATP) synthesis, the fundamental energy currency of cells. This study used Computer-Aided Drug Design approaches to identify potential inhibitors of *C*. *koseri* ATP synthase. SWISS-MODEL was used to predict the 3D structure of *C*. *koseri* ATP synthase. A ligand-based pharmacophore model was developed using chemical features of ampicillin. Following ligand-based virtual screening across nine databases, the 2043 screened hits were docked to the ATP synthase active site using the standard precision mode of the glide tool. Based on their binding affinities, the top ten compounds were selected for additional investigation. The binding affinities of the chosen compounds ranged from -10.021 to -8.452 kcal/mol. The top four compounds (PubChem-25230613, PubChem-74936833, CHEMBL263035, PubChem-44208924) with the best ADMET characteristics and binding modes were chosen. Thus, the feasible binding mechanisms of the selected compounds were subjected to stability analysis using the MD Simulation study, which revealed the compounds’ stability as potent inhibitors within the protein binding pocket. This computational approach provides important insights into the rational design of novel therapeutics and emphasizes the importance of targeting essential metabolic pathways when combating antibiotic-resistant pathogens. Future experimental validation and optimization of the identified inhibitors is required to determine their efficacy and safety profiles for clinical use.

## 1. Introduction

*Citrobacter koseri* (formerly *Citrobacter diversus*) is a bacterium in the Citrobacter genus. *C*. *koseri* is widely found in the human gastrointestinal tract, where it does no harm. However, in certain circumstances, it can become opportunistic and cause infections, particularly in people with weakened immune systems [1, 2]. *C*. *koseri* has been associated with a variety of diseases, including urinary tract infections, respiratory tract infections, meningitis, and bloodstream infections (bacteremia) [3–5]. Maintaining appropriate hygiene standards is usually necessary for the prevention of *C*. *koseri* infections, particularly in healthcare settings where the bacteria may be a source of hospital-acquired infections. This covers routine hand washing, keeping medical equipment clean, and infection control methods [6, 7].

*C*. *koseri* has recently gained resistance to a number of antibiotics, including third-generation cephalosporins, beta-lactamases, aminoglycosides, and quinolones [8, 9]. Infections caused by *C*. *koseri* require a range of potent drugs to be effectively treated, particularly if the bacteria exhibit resistance to widely prescribed antibiotics. Healthcare providers can customize a patient’s therapy to meet their unique needs by having access to a variety of treatment options [7, 10–12]. Hence, having effective anti-*C*. *koseri* drugs is critical for patient treatment, public health, and the ongoing fight against antibiotic resistance.

*C*. *koseri* possesses an ATP (Adenosine Triphosphate) synthase enzyme, which is essential for energy synthesis in many living species. ATP synthase is a complicated enzyme found in eukaryotic cells’ inner mitochondrial membranes and prokaryotic cells’ cytoplasmic membranes. It is essential for cellular respiration because it catalyzes the production of adenosine triphosphate (ATP), the cell’s basic energy currency [13]. ATP synthase is frequently referred to as the "molecular machine" that generates ATP. It does this by making use of the proton gradient that runs through the cytoplasmic or inner mitochondrial membrane. This process is required for the bacterium’s metabolic activities and growth [14–16].

Inhibiting ATP synthase is a potential target for developing antibiotics. By disrupting the energy production in the bacterial cell, you can weaken the bacterium and make it more susceptible to the host’s immune system and other antibiotics. ATP synthase inhibitors could provide a novel method for fighting antibiotic resistance, particularly if they target a different mechanism than existing antibiotics. Some people may be allergic to or have harmful responses to existing antibiotics. For individuals who are unable to tolerate standard antibiotics, having an alternate therapy option such as an ATP synthase inhibitor can be critical [13, 17].

Utilizing computational methods has become a new tool in the fight to create therapeutic compounds with the greatest possible number of pharmacophoric characteristics. Through chemical synthesis, *in vitro* testing, and *in vivo* testing, these strategies hope to cut down on the time and resources needed. The primary goal of computer-aided or *in silico* drug design, or CADD, which seeks to speed and simplify the drug discovery process, is to determine which lead candidate is most likely to be a good candidate for biological testing [18–20]. In this study, various computational techniques, such as virtual screening, pharmacophore modeling, molecular docking, and molecular dynamics simulation were used to identify potential *C*. *koseri* ATP synthase inhibitors.

## 2. Methodology

### 2.1 Structure prediction

The 3D structure of *C*. *koseri* ATP synthase was predicted using SWISS-MODEL [21], with the structure of E. coli ATP synthase (PDB ID: 6OQR) as a template, given the lack of an experimental structure for C. koseri ATP synthase [22, 23]. Additionally, an ab initio model was obtained from AlphaFold [23] (ID: AF-A8ACN6-F1). The amino acid sequence for this analysis was sourced from the UniProt database [24] using the RefSeq ID: WP_012000829.1.

### 2.2 Structure validation

The preliminary structural evaluation of the predicted protein models was conducted using the VERIFY and ERRAT [25] tools. A model of high quality should have an ERRAT score of around 91%. The best model, based on these assessments, was the one generated by SWISS-MODEL. Further quality assessment of the top model from the AlphaFold model and the SWISS-MODEL was performed using MolProbity (http://molprobity.biochem.duke.edu/index.php) for validating the quality of nucleic acid and protein structures. With respect to factors like clash, rotamer, and Ramachandran scores, the MolProbity score offers a unified measure of model quality. This comprehensive evaluation helped ensure the reliability and quality of the predicted protein structures.

### 2.3 Active site prediction

The Computed Atlas of Surface Topography of Proteins (CASTp) 3.0 tool was used to forecast the active sites of the modeled ATP synthase structure [26]. CASTp is a valuable resource for identifying and characterizing the active sites and pockets on protein structures, providing insights into potential binding sites, substrate interaction regions, and other functionally relevant areas within the protein.

### 2.4 Pharmacophore modelling and virtual screening

All the key structural components of compounds that are biologically active are included in a chemical template called a pharmacophore model. A pharmacophore model is created using the structural characteristics of an active compound, and it is subsequently processed to perform chemical database screening [27]. A ligand-based pharmacophore model was developed using the chemical features of ampicillin by utilizing the Pharmit server [28, 29]. The server offers a protocol to screen the chemical databases based on the developed pharmacophoric features. The chemical structure of ampicillin was used to develop the pharmacophore model based on its interactions with ATP synthase predicted binding sites. Four pharmacophoric features of ampicillin i.e., hydrogen bond acceptor, hydrogen bond donor, aromatic, and hydrophobic were used to generate the model for virtual screening. For the virtual screening, the following databases were explored: ZINC, LabNetwork, PubChem, MolPort, MCULE-Ultimate, MCULE, ChemSpace, ChemDiv, and CHEMBL.

### 2.5 Ligand preparation

From the pharmacophore-based virtual screening, 2043 hits were produced, and LigPrep prepared them for docking [30]. The OPLS_2005 forcefield was used to create the minimized energy conformers of each hit for use in the docking step [31].

### 2.6 Molecular docking

The molecular docking of the prepared hits was conducted against the ATP synthase receptor. The validated structure of ATP synthase was ready for docking using Protein Preparation Wizard [32]. The process of preparing the protein involved multiple steps. Zero-bond orders were established, disulfide bonds were produced, and zero-order metal bonds were assigned. Furthermore, the protein structures were added with hydrogen and then adjusted to pH 7.0 by calculating the ionizable groups’ pKa values using the PROPKA program [33]. Finally, the protein energy was minimized by using the OPLS_2005 forcefield. After protein preparation, a 3D grid was generated at X, Y, and Z coordinates, with the values of -12.13, 5.63, and 19.45, respectively. Finally, the SP model of the glide tool was used to dock the prepared ligands to the protein [34]. The glide scores of the docked ligands were analyzed and selected.

### 2.7 MD simulation

The protein ligand stability was examined by subjecting the binding modes of the chosen compounds and the control to a 300 ns MD simulation. For every compound, a cubic box measuring 10 Å was filled with TIP3P water molecules to dissolve the protein-ligand complex [35]. Na^+^ and Cl^-^ counterions were added to the system to neutralize it. To eliminate steric clashes, the system was minimized using the steepest decent technique for 5000 steps after neutralization. Following minimization, the systems were set up for equilibration at NVT and NPT ensemble for 50,000 and 100,000 steps, respectively, at 310 K temperature [36]. The simulation was run with Parrinello-Rahman and Berendsen thermostat algorithms to keep the pressure (1 atm) and temperature (310 K) constant. By setting the time at τ P = 2.0 ps and τ T = 0.1 ps, the system was relaxed. The LINCS algorithm was utilized to maintain the optimal bond lengths of hydrogen atoms [37], and Verlet was utilized to calculate the non-bonded interactions [38]. The electrostatic interactions above the short-range cutoff were computed using the particle mesh Ewald method [39]. In the x, y, and z dimensions, the periodic boundary conditions were applied, and a production run was conducted on the system. The trajectories were saved and examined using the BIO3D package and Gromacs commands [40]. The CHARMM36 forcefield and the Gromacs simulation package were used to run the simulation [41].

### 2.9 MMGBSA

The binding free energies of the complexes were calculated by employing the MMGBSA method. The binding free energies were determined by subtracting the receptor and ligand free energy from the complex free energy (Eq 1). Further, the entropy changes were calculated by employing Eq 2. Moreover, the residue binding energy decomposition was estimated to find the key interacting residues. Eq 3 was used for the calculation of binding free energy decomposition [42].


$$\Delta G_{Bind}=\Delta G_{complex}‐(\Delta G_{protein}+\Delta G_{ligand})$$ (1)

$$T\Delta S=T(\Delta S_{trans}+\Delta S_{rot}+\Delta S_{vib})$$ (2)

$$\Delta G_{Inhibitor‐residue}=\Delta G_{vdW}+\Delta G_{ele}+\Delta G_{ele,\;sol}+\Delta G_{nonpol,\;sol}$$ (3)

### 2.10 ADMET analysis

The higher rate of drug erosion is often attributed to issues related to toxicity and poor pharmacokinetics [43]. To address these challenges, ADMET properties are predicted to assess the pharmacokinetic properties and toxicity risks associated with potential drug candidates [44]. The ADMET properties were predicted by QikProp tool [42]. Additionally, the drug likeness was predicted by the miDruglikeness webserver [45].

## 3. Results

### 3.1 Structure validation

The evaluation of the ATP synthase models’ structures using the SWISS-MODEL and AlphaFold models revealed that, according to different evaluation scores, the SWISS-MODEL structure performed better than the AlphaFold model. The SWISS-MODEL structure achieved an impressive VERIFY score of 81.22% and an ERRAT score of 97.89% (Table 1). Moreover, the SWISS-MODEL structure showed its superiority even after undergoing additional structural evaluation in contrast to the AlphaFold model (Table 2). Merely 0.22% of the amino acids in the SWISS-MODEL structure were classified as Ramachandran outliers, while 97.82% of the amino acids were found in the Ramachandran favored region. By contrast, the AlphaFold model contained the same 0.22% of Ramachandran outliers and 96.93% of its amino acids in the Ramachandran favored region.

**Table 1:** The assessment of ATP synthase model by ERRAT and VERIFY.

Model	ERRAT (%)	VERIFY (%)
SWISS-MODEL	97.89	81.22% (Pass)
AlphaFold	97.29	70.65 (Fail)

**Table 2:** The comparison of MolProbity parameters of predicted structures.

	Parameters	SWISS-MODEL		AlphaFold		
**All-Atom** **Contacts**	Clash score	0.43 (99^th^ percentile)		1.84(99^th^ percentile)		Goals
**Protein geometry**	Poor rotamers	10	2.63%	0	0.00%	<0.3%
	Favored rotamers	349	91.84%	379	99.48%	>98%
	Ramachandran outliers	1	0.22%	1	0.22%	<0.05%
	Ramachandran favored	442	97.82%	448	96.93%	>98%
	Rama distribution Z-score	0.04± 0.38		0.83 ± 0.39		Z score < 2
	MolProbity score	1.15		0.99		0Å - 99Å
	Cβ deviations >0.25Å	5	1.21%	0	0.00%	0
	Bad bonds	0 / 3578	0.00%	0 / 3591	0.00%	0%
	Bad angles	25 / 4844	0.52%	11 / 4861	0.23%	<0.1%
**Peptide Omegas**	Cis Prolines	1 / 20	5.00%	1 / 20	5.00%	≤1 or ≤5%
**Low-resolution Criteria**	CaBLAM outliers	4	0.9%	3	0.7%	<1.0%
	CA Geometry outliers	0	0.00%	0	0.00%	<0.5%
**Additional validations**	Chiral volume outliers	0/553		0/555		

### 3.2 Pharmacophore modelling and virtual screening

**Fig 1:**
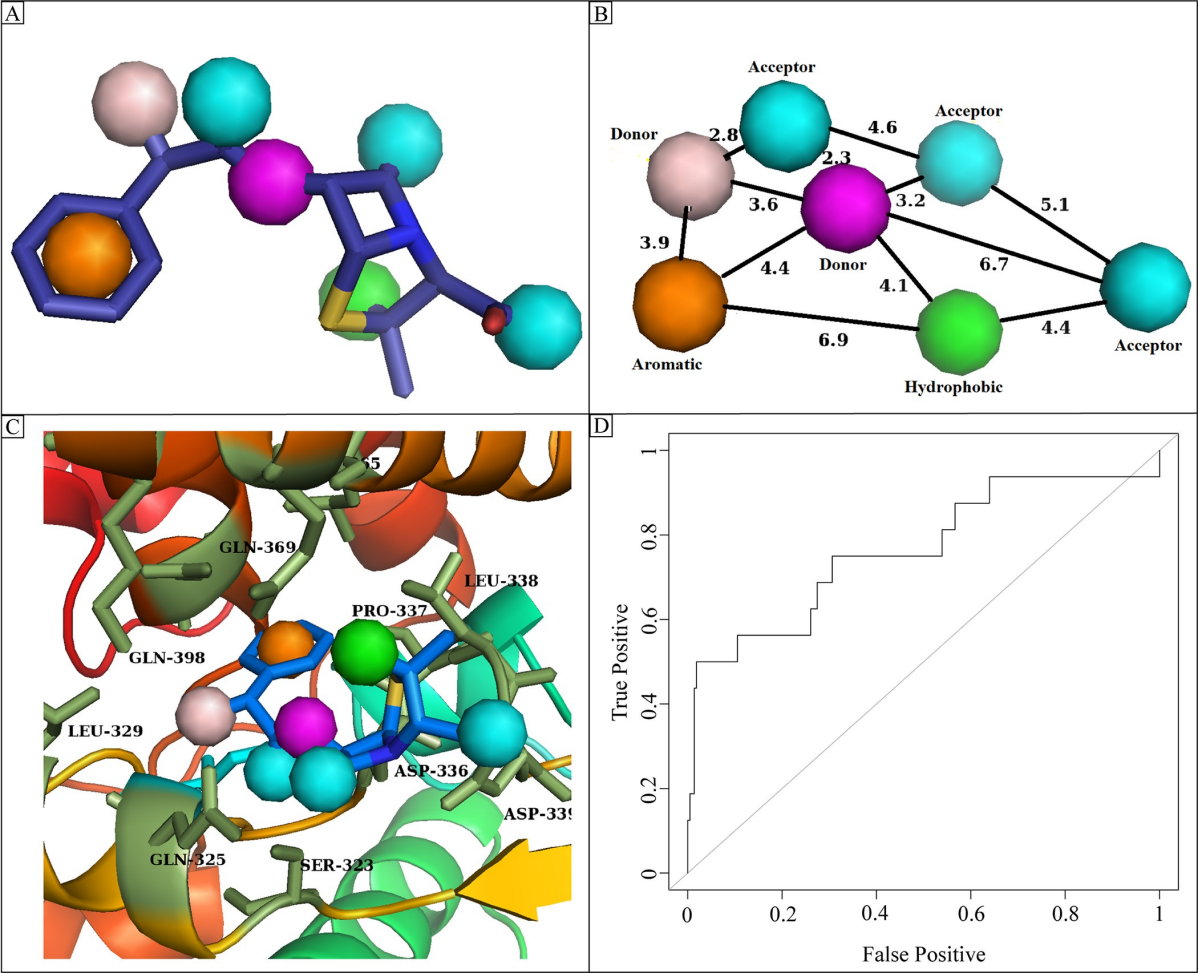
(A) The seven-point pharmacophore query model generated by Pharmit server. The green sphere shows hydrophobic group, orange shows the aromatic ring, purple and skin sphere shows the Hydrogen bond donors while cyan spheres show the hydrogen bond acceptor. (B) The distances among the features of seven-point pharmacophore model. (C) The representation of developed pharmacophore model in protein pocket. (D) The ROC curve of the model with an AUC value of 0.76.

The pharmacophoric features of ampicillin involved in the molecular interactions with the ATP synthase protein were used to develop the pharmacophore query model. There was a total of seven features that were used to generate the query model (Fig 1A). The distances of the features of seven-point pharmacophore model DDAAAHR were measured, and it was observed that distance between aromatic and hydrophobic group was 6.9 Å, the distance between donor and donor groups was 3.6 Å, similarly, the highest distance between two acceptor groups was 5.1 Å and lowest distance was 4.6 Å. The lowest distance among all features was observed between donor and acceptor group which was 2.3 Å (Fig 1B). Further, the pharmacophore model was represented within the active site of the protein to analyze the interacting residues of protein with the pharmacophore (Fig 1C). The X, Z, and Y coordinates of the seven-point pharmacophoric features are shown in Table 3. Prior to the screening, the pharmacophore model was validated by calculating the Area under curve (AUC) values by utilizing R code for receiver operating characteristic (ROC) curve. A data set of active and decoys was used for the validation of model and the ROC curve for the generated query model showed that the model had an ability to distinguish active compounds from decoys with an AUC value of 0.76 (Fig 1D). The validated pharmacophore model was then subjected for the virtual screening of eight databases. The screening compounds that match with the seven-point pharmacophore model with a minimum RMSD of 0.5 Å were selected for further studies (Table 4). There were a total of 2043 hits collectively obtained from the nine databases. Among these hits, the PubChem database produced the highest number of hits.

**Table 3:** The pharmacophoric features their coordinates, generated by Pharmit server.

Pharmacophoric Features	X	Y	Z	Radius
Hydrogen Donor	-10.68	5.43	19.98	1
Hydrogen Donor	-9.68	2.68	22.05	1
Hydrogen Acceptor	-9.79	8.49	20.37	1
Hydrogen Acceptor	-12.92	11.27	17.45	1
Hydrogen Acceptor	-8.88	4.08	19.75	1
Hydrophobic	-14.05	8.46	19.34	1
Aromatic	-12.4	1.4	19.57	1

**Table 4:** The generated hits from each database based on ligand-based virtual screening.

Sr.	Databases	Hits
1	CHEMBL	195
2	Chemspace	3
3	MCULE	60
4	MCULE-Ultimate	3
5	Molprot	37
6	PubChem	1651
7	LabNetwork	29
8	ZINC	65
	Total	2043

### 3.3 Molecular docking

**Fig 2:**
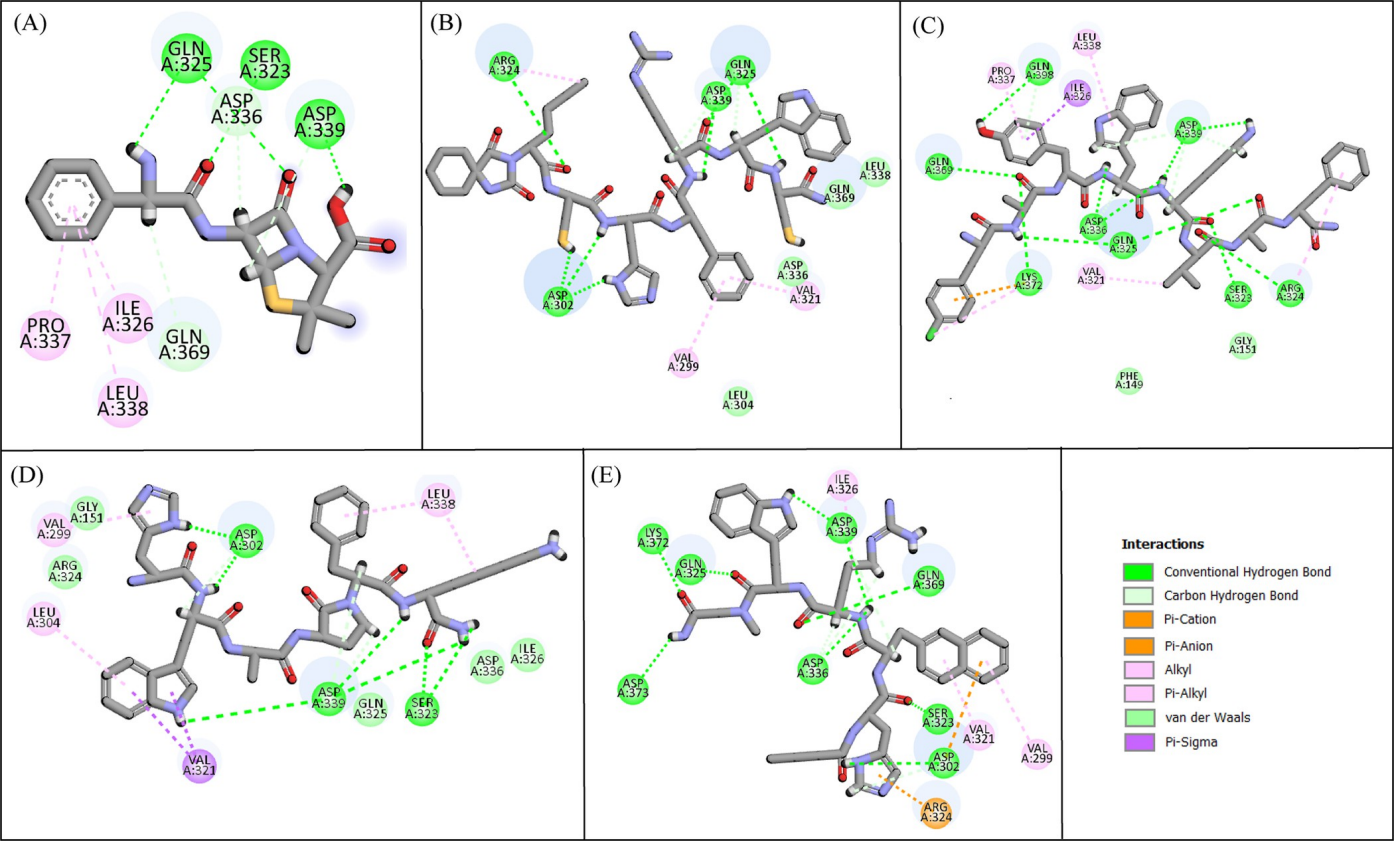
The molecular interactions of the top 4 compounds. (A) Control, (B) PubChem-25230613, (C) CHEMBL263035, (D) PubChem-74936833, (E) PubChem-44208924.

The optimized ATP synthase receptor was docked to the hit compounds produced during virtual screening, predicting binding affinities with the glide tool’s standard precision mode. Ten compounds were chosen for additional examination based on their binding affinities (Table 5). The compounds had binding affinities ranging from -10.021 to -8.452 kcal/mol, better than the binding affinity of Control (-5.177 kcal/mol). The binding affinities of the chosen compounds indicated that they have a probability of inhibiting the function of ATP synthase. The Discover Studio client tool was utilized to assess the molecular interactions among the selected hits and the binding pocket of the ATP synthase receptor. The observed interactions involved: carbon hydrogen bond, van der Waal interactions, conventional hydrogen bond, pi-sulfur, amide pi-stacked, halogen, and alkyl interactions. The binding affinities and docking scores of each of the best candidate compounds are determined by these interactions. Notably, the overall strength of the resulting complex is significantly influenced by the formation of intermolecular hydrogen bonds between the amino acid and the ligand within the active sites. Consequently, these interactions consistently enhance the docking results [46]. The **Control** made three conventional hydrogen bonds with Gln325, Ser323, Asp339, one carbon hydrogen bond with Gln369, and three alkyl interactions with Ile326, Pro337, and Leu338 (Fig 2A). **PubChem-25230613** formed four van der Waal interactions with Leu304, Asp336, Gln369, Leu338, four conventional hydrogen bonds with Arg324, Asp302, Asp339, Gln325, and two alkyl interactions with Val299 and Val321 (Fig 2B). **CHEMBL263035** made two van der Waal interactions with Phe149, Gly151, eight conventional hydrogen bonds with Gln369, Gln398, Lys372, Asp336, Gln325, Asp339, Ser323, Arg324, one Pi-Cation with Lys372, one Pi-Sigma interaction with Ile326, and three alkyl interactions with Pro337, Leu338, Val321 (Fig 2C). Similarly, **PubChem-74936833** formed five van der Waal interactions with Gln325, Asp336, Ile326, Gly151, Arg324, three conventional hydrogen bonds with Asp302, Asp339, Ser323, one Pi-Sigma with Val321, and three alkyl interactions with Val299, Leu304, Leu338 (Fig 2D). Lastly, **PubChem-44208924** made eight conventional hydrogen bonds with Lys372, Asp373, Gln325, Asp339, Asp336, Gln369, Ser323, Asp302, one carbon hydrogen bond with Asp339, one Pi-Cation interaction with Arg324, and three alkyl interactions with Ile326, Val321, Val299 (Fig 2E).

**Table 5:** The binding affinities of the selected compounds along with their structures and molecular interactions.

Sr.	Compound code	Glide score	Interactions
1	PubChem-25230613 (PubChem ID)	-10.0	**Van der Waal**: Leu304, Asp336, Gln369, Leu338 **Conventional Hydrogen Bond:** Arg324, Asp302, Asp339, Gln325 **Carbon Hydrogen Bond:** Gln325 **Alkyl:** Val299, Val321
2	PubChem-74936833 (PubChem ID)	-9.7	**Van der Waal**: Gln325, Asp336, Ile326, Gly151, Arg324 **Conventional Hydrogen Bond:** Asp302, Asp339, Ser323 **Pi-Sigma:** Val321 **Alkyl:** Val299, Leu304, Leu338
3	CHEMBL263035 (CHEMBL ID)	-9.4	**Van der Waal:** Phe149, Gly151 **Conventional Hydrogen Bond:** Gln369, Gln398, Lys372, Asp336, Gln325, Asp339, Ser323, Arg324 **Pi-Cation:** Lys372 **Pi-Sigma:** Ile326 **Alkyl:** Pro337, Leu338, Val321
4	PubChem-44208924 (PubChem ID)	-9.3	**Conventional Hydrogen Bond:** Lys372, Asp373, Gln325, Asp339, Asp336, Gln369, Ser323, Asp302 **Carbon Hydrogen Bond:** Asp339 **Pi-Cation:** Arg324 **Alkyl:** Ile326, Val321, Val299
5	CHEMBL4078128 (CHEMBL ID)	-9.2	**Conventional Hydrogen Bond:** Glu370, Asp339, Gln398, Asp336, Asp302 **Carbon Hydrogen Bond:** Gln325 **Pi-Sigma:** Val321 **Alkyl:** Ile326, Pro337, Leu304, Val299
6	ZINC000169306132 (ZINC ID)	-9.1	**Van der Waal:** Val299, Ile326 **Conventional Hydrogen Bond:** Ala301, Tyr298, Asp302, Arg324, Asp339, Gln325, Lys372 **Pi-Sigma:** Val321 **Alkyl:** Phe149, Leu304
7	PubChem-10418690 (PubChem ID)	-9.0	**Van der Waal:** Gln369, Pro337, Ser323 **Conventional Hydrogen Bond:** Lys372, Asp373, Asp339, Gln325, Asp302, Arg324, Val299 **Carbon Hydrogen Bond:** Asp336, Gly150 **Alkyl:** Leu338, Ile326, Val321, Tyr298
8	PubChem-44240874 (PubChem ID)	-8.6	**Van der Waal:** Val299, Gln369 **Conventional Hydrogen Bond**: Asp302, Asp339, Gln325, Asp336, Ser323, Val335, Gln398, Lys372 **Pi-Sigma:** Val321 **Alkyl:** Ile326
9	PubChem-71695663 (PubChem ID)	-8.5	**Conventional Hydrogen Bond:** Asp302, Ser323, Asp339, Gln325, Arg324, Glu370 **Carbon Hydrogen Bond:** Gly151 **Alkyl:** Ile326, Leu338
10	PubChem-25119387 (PubChem ID)	-8.4	**Van der Waal:** Gln325, Ile326, Lys372, Arg324, Val321, Val299 **Conventional Hydrogen Bond:** Asp336, Asp302, Asp339, Thr319 **Alkyl:** Leu304, Leu338, Phe313
11	Control (Ampicillin)	-5.1	**Conventional Hydrogen Bond:** Ser323, Gln325, Asp339 **Carbon Hydrogen Bond:** Gln369 **Alkyl:** Ile326, Pro337, Leu338

### 3.4 Binding pose analysis

**Fig 3:**
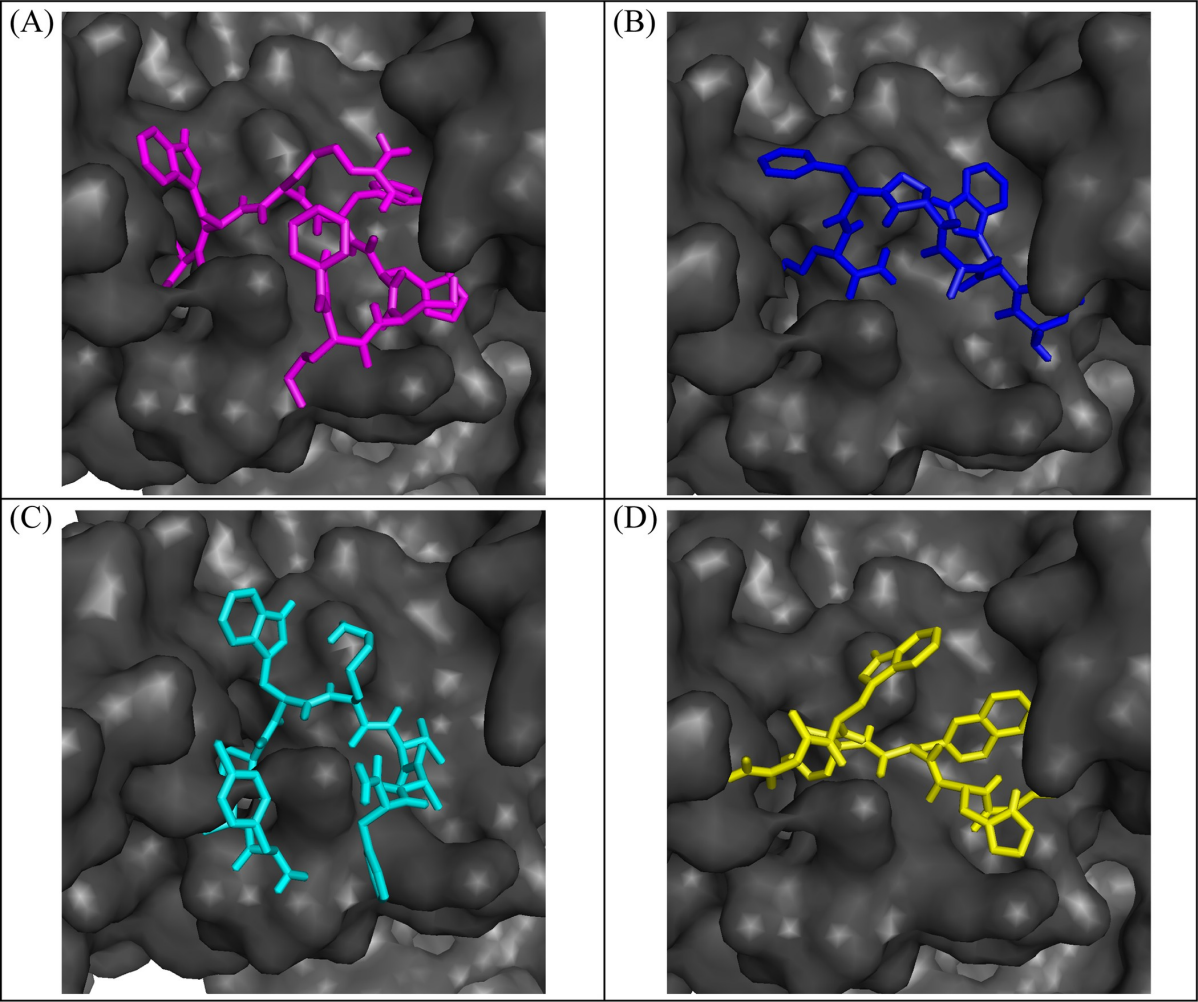
The plausible binding modes of the top four compounds. (A) PubChem-25230613 (Magenta sticks), (B) PubChem-74936833 (Blue sticks), (C) CHEMBL263035 (Cyan sticks), (D) PubChem-44208924 (Yellow sticks).

Following the examination of molecular interactions, the binding modes for the top four compounds were examined. The analysis showed that the docked compounds occupied the predicted binding sites accurately (Fig 3). Thus, the MD simulation study was used to analyze the stability of the binding modes of the chosen compounds.

### 3.5 MD simulation

#### 3.5.1 RMSD

**Fig 4:**
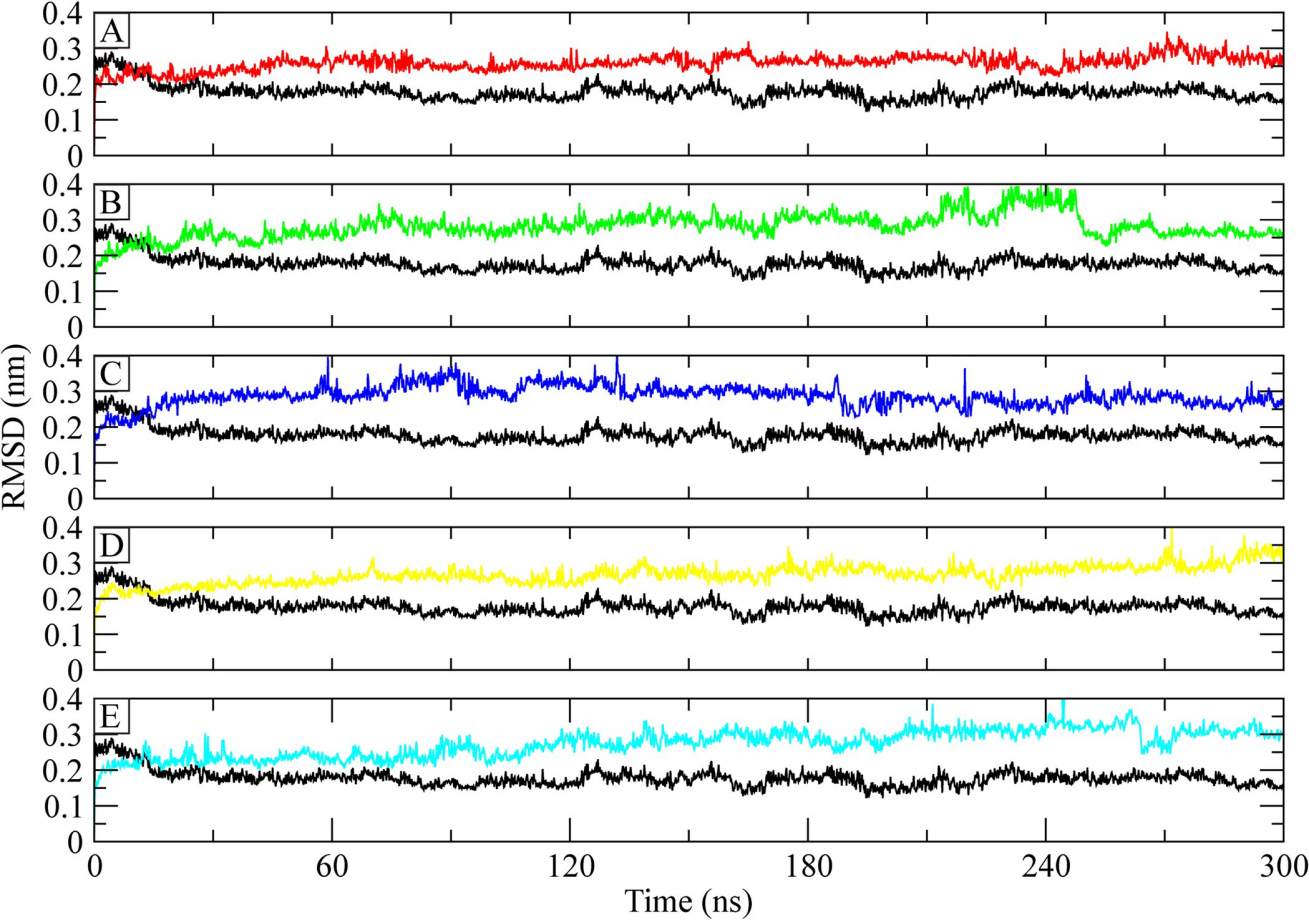
The RMSD of C-alpha atoms of ATP synthase complex with control and selected hits. The RMSD of the apo protein is indicated by black color. (A) Control, (B) CHEMBL263035, (C) PubChem-25230613, (D) PubChem-44208924, (E) PubChem-74936833.

The stability of the protein-ligand complex was determined by computing the RMSD of the C-alpha atoms of the apo ATP synthase protein and its complexes with control and chosen hits [47]. The RMSD of apo protein was around 0.25 nm at the start of simulation which gradually decreased to 0.2 nm at 30 ns. After 30 ns, the RMSD attained stability in this range till the end of simulation. On the other hand, the RMSD of **control** remained in the range of 0.2–0.25 nm till 30 ns and then progressively increased to 0.3 nm at 60 ns. It dropped to 0.25 nm again at 150 ns and then attained stability in the range of 0.25 nm to 240 ns, the RMSD reached 0.35 nm in the last part of simulation (Fig 4A). The RMSD of the **CHEMBL263035** complex showed higher values than **Control** as it reached 0.3 nm at 30 ns and remained in this range till 180 ns, then it increased to 0.4 nm at 230 ns. The RMSD values dropped to 0.25 nm at 260 ns and then maintained this range towards the end of simulation (Fig 4B). The RMSD of **PubChem-25230613** showed a similar trend as it was higher than control and reached to 0.4 nm at 60 ns and deviated in this range till 130 ns, but it started to drop to 0.25 nm after 180 ns and then decreased to 0.25 nm at 240 ns and then maintained this range till 300 ns (Fig 4C). The RMSD plot of **PubChem-44208924** showed a similar trend to the control RMSD, remained in the range of 0.25 nm till 250 ns and then reached to 0.3 nm towards the end of simulation (Fig 4D). Lastly, the RMSD **PubChem-74936833** showed deviations in the range of 0.3 nm till 100 ns and then increased to 0.35 nm at 120 ns. It reached 0.4 nm at 250 ns, but it dropped to 0.3 nm around 270 ns and then maintained this range till the end of simulation (Fig 4E). According to the RMSD analysis, the hits were found to be stably bound to the protein throughout the 300 ns simulation.

#### 3.5.2 RMSF

**Fig 5:**
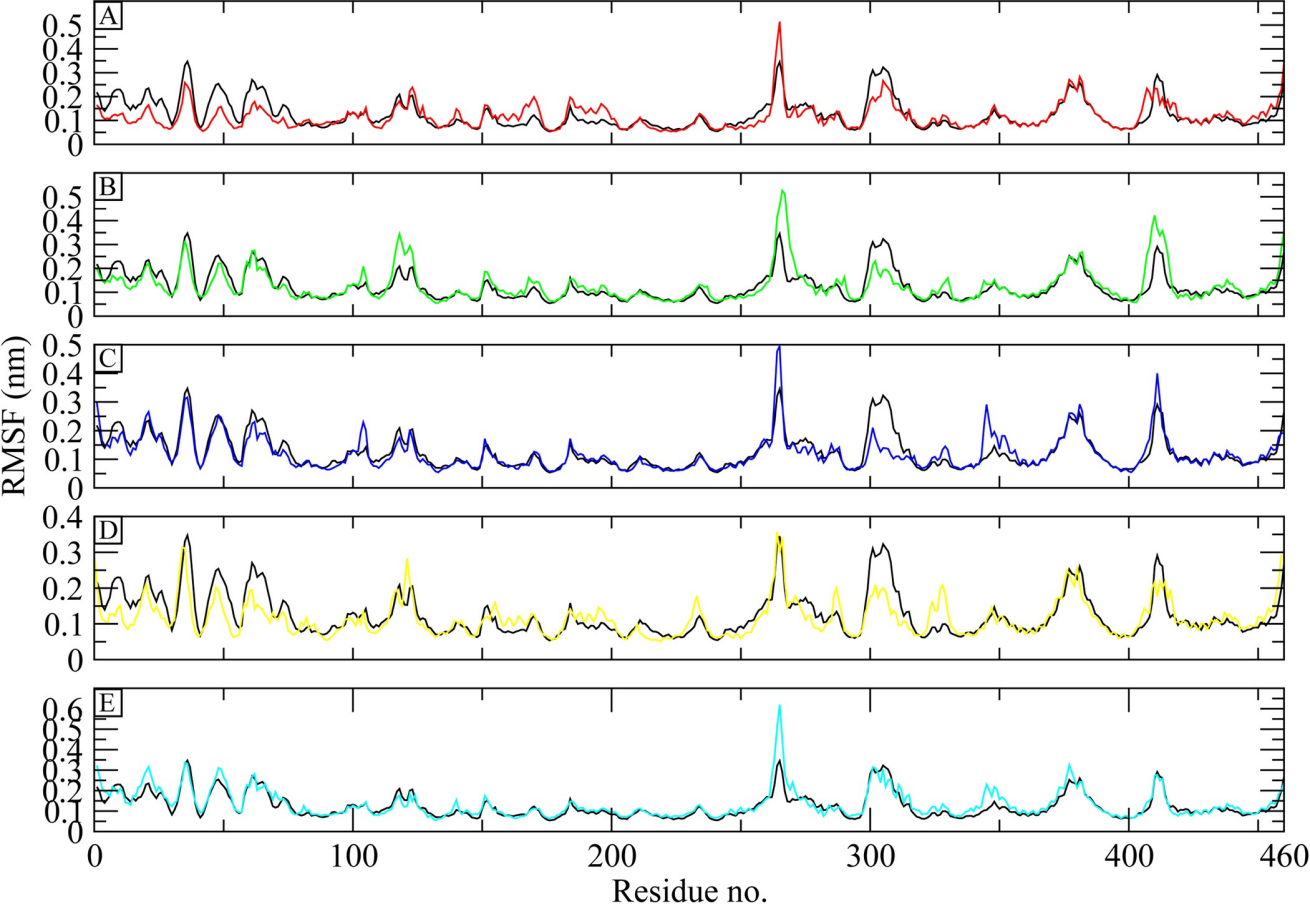
The residual fluctuations of the apo ATP synthase receptor and complexes. The Apo receptor is shown with black color. (A) Control, (B) CHEMBL263035, (C) PubChem-25230613, (D) PubChem-44208924, (E) PubChem-74936833.

Root Mean Square Fluctuations (RMSF) of protein residues were computed and examined in order to examine the dynamic behavior of the protein while bound to the Control and chosen hits by comparing them with the flexibility of apo structure [48]. The flexible portions of the protein, or loops, are indicated by high RMSF values, whereas the presence of compact secondary structures is demonstrated by lower values. The RMSF plots of the Control and hit complexes showed a similar trend in terms of fluctuations, i.e., most of the residues remained rigid with RMSF values less than 0.2 nm. On the other hand, residues ranging from 260 to 280, 300 to 320, 350 to 360, 380 to 400, and 410 to 420 showed higher RMSF values, indicating the presence of loop regions. Some of the residues near the active sites showed high RMSF values, for example the residues 300 to 320, and 410 to 420 reside near the active site, but these residues show higher fluctuations in the apo structure as well (Fig 5). Overall, protein did not show major fluctuations, indicating that the bound ligand did not exert major confirmational changes to the protein structure during the simulation.

#### 3.5.3 Radius of Gyration (Rg)

**Fig 6:**
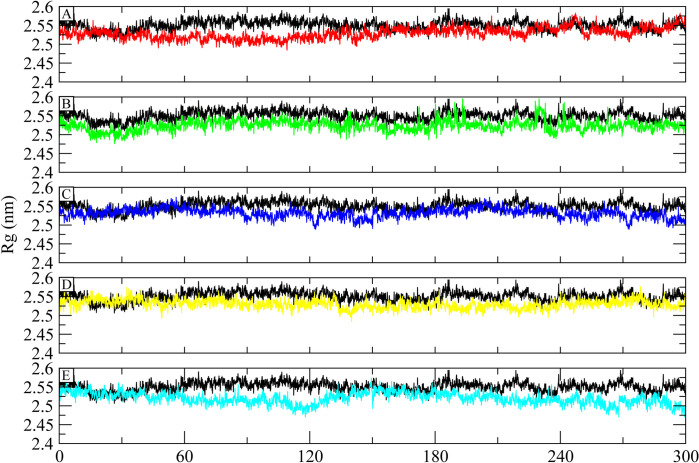
The Rg analysis of the apo ATP synthase and its complexes. The Apo receptor is shown with black color. (A) Control, (B) CHEMBL263035, (C) PubChem-25230613, (D) PubChem-44208924, (E) PubChem-74936833.

The structural compactness of the apo ATP Synthase and its complexes bound to the control and chosen hits was evaluated using Radius of Gyration (Rg) analysis. Higher Rg values depict events that are unfolding during simulation, whereas lower Rg values indicate how compact the structure is. The Rg values reached stability in the range of 2.5 to 2.55 nm and remained in this range throughout the simulation. Interestingly, the Rg values of the complexes were lower than the apo structure so it can be deducted that when bound to these compounds, the protein structure remained compacted and did not experience any unfolding events during simulation (Fig 6).

#### 3.5.4. SASA

**Fig 7:**
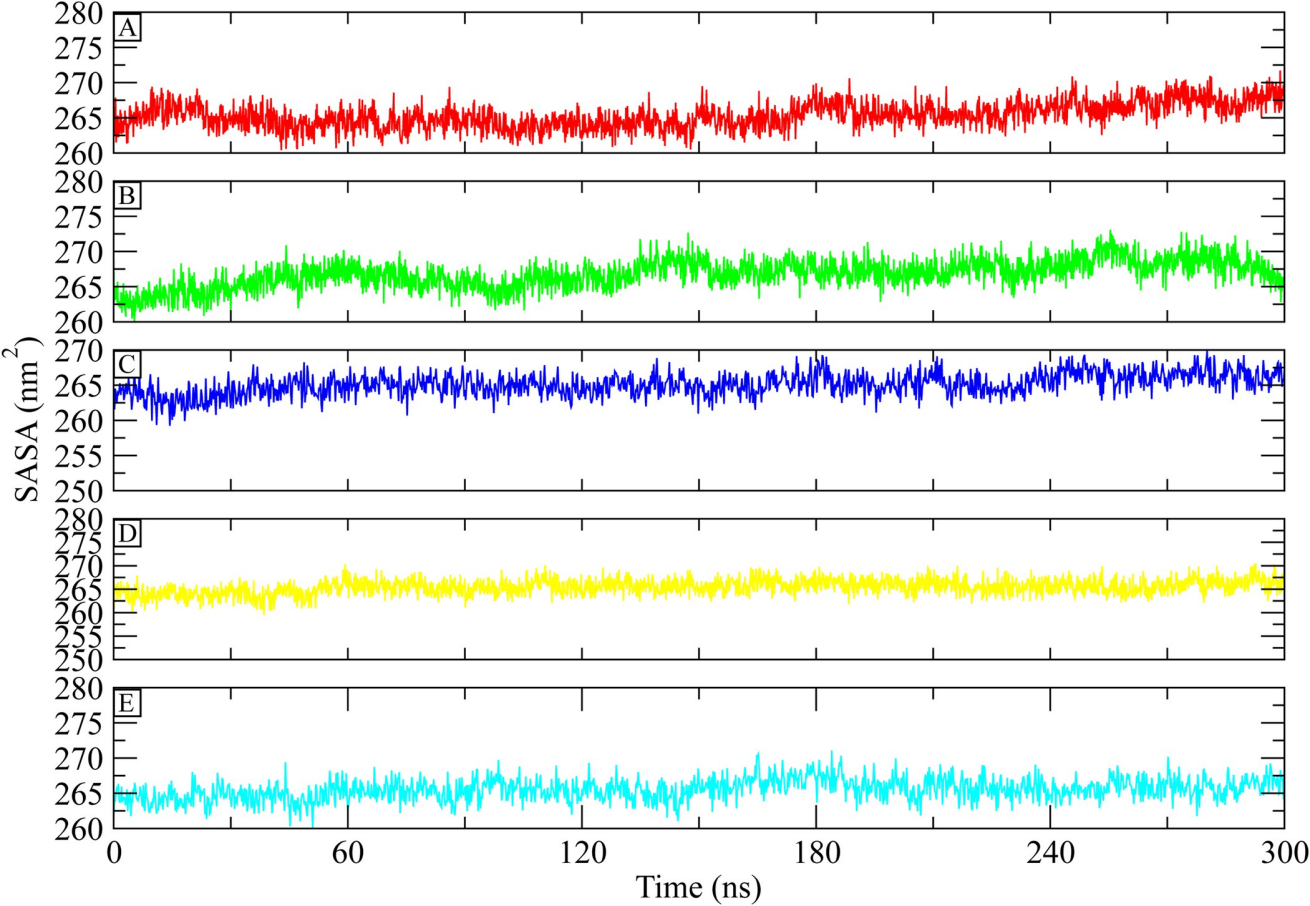
The SASA plots of the ATP synthase complexes with control and selected compounds. (A) Control, (B) CHEMBL263035, (C) PubChem-25230613, (D) PubChem-44208924, (E) PubChem-74936833.

The solvent accessible area of the protein during simulation was estimated using “Solvent Accessible Surface Area” (SASA) analysis, which also looked for conformational changes. After the simulation, the protein’s SASA value was found to have started at about 265 nm^2^, and it stayed in the range of 262–267 nm^2^ (Fig 7). According to the SASA values, there were no conformational changes to the protein structure during the simulation.

#### 3.5.5. Hydrogen bonding

**Fig 8:**
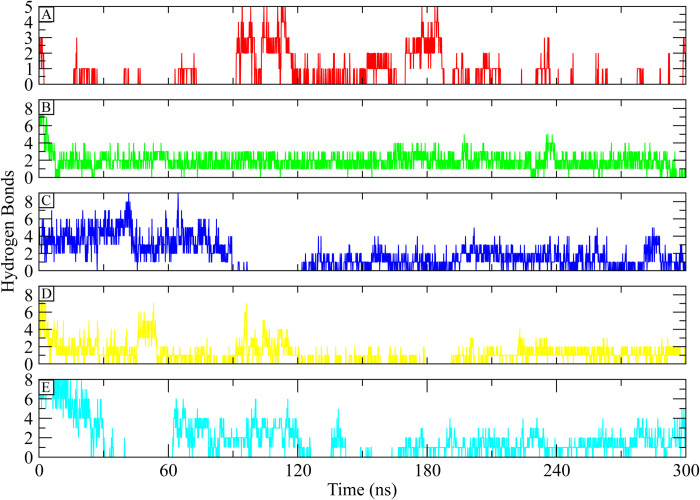
The hydrogen bonds formation between ATP Synthase and selected compounds during 300 ns simulation. (A) Control, (B) CHEMBL263035, (C) PubChem-25230613, (D) PubChem-44208924, (E) PubChem-74936833.

The stability of the protein-ligand complex is significantly influenced by hydrogen bonding. The hydrogen bonds between the ligand and the residues of the active site were therefore computed. The hydrogen bonding plots indicate that **Control** made a minimum of two hydrogen bonds up to 100 ns while at some frames, no hydrogen bond was observed. After 100 ns, the number of hydrogen bonds increased to 4 and maintained 2 to 4 hydrogen bonds till 220 ns and towards the end of simulation, hydrogen bonds were missed at some frames (Fig 8A). **CHEMBL263035** made at least three hydrogen bonds throughout the simulation (Fig 8B). **PubChem-25230613** showed up to 6 hydrogen bonds till 100 ns and then missed at some frames, after 120 ns, minimum 2 hydrogen bonds were observed till the end of simulation (Fig 8C). In the case of **PubChem-44208924** complex, minimum two hydrogen bonds were observed throughout the simulation (Fig 8D), while in **PubChem-74936833** complex, missing hydrogen bonds were observed during 40 to 60 ns and 150 to 160 ns, while for the remaining time, the complex maintained minimum two hydrogen bonds (Fig 8E). The analysis of hydrogen bonds showed that the hits formed more bonds than the control, indicating that they formed a more stable complex than the **Control**.

#### 3.5.6. PCA

**Fig 9:**
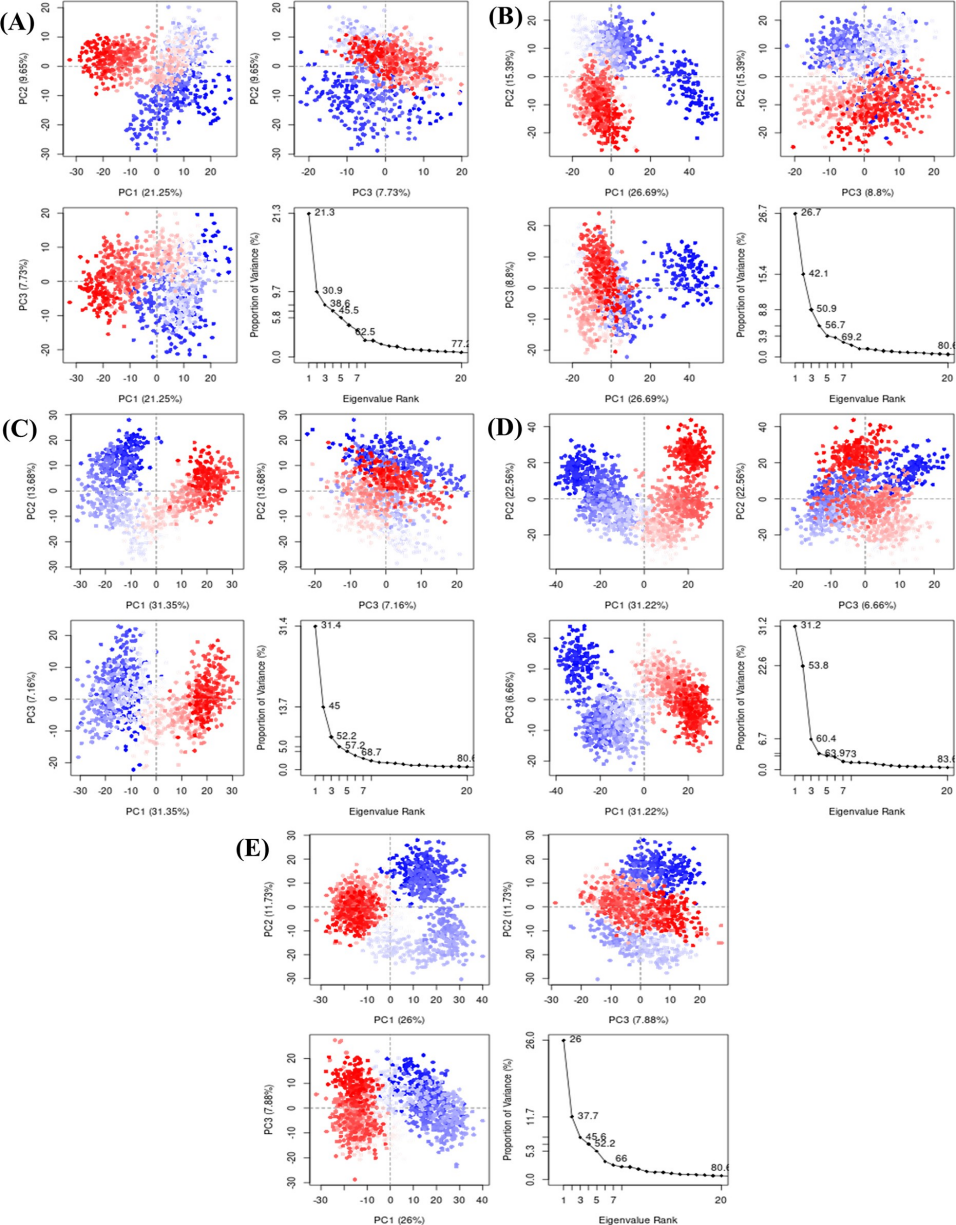
Principal component analysis of the control and hits complexes. (A) PCA of Control with total variance of 38.63% in first three PCs. (B) PCA of PubChem-25230613 with total variance of 50.88%. (C) PCA of CHEMBL-263035 with total variance of 52.19%. (D) PCA of PubChem-74936833 with total variance of 60.44%. (E) PCA of PubChem-44208924 with total variance of 45.61%.

Principal Component Analysis determines the collective motions of proteins in different hyperspaces and was used to examine the dynamic behavior of the proteins. The eigen value, which depicts the dynamic motions, was plotted against the variance proportion. Since they covered the main fluctuations, only three PCs were plotted. It is evident from the PCA plot of the **Control** that PC1 displayed the greatest variation (21.25%) compared to the others. The variability was shown by PCs 2 and 3, which were 9.65% and 7.73%, respectively (Fig 9A). Similarly, the PC1 in **PubChem-25230613** showed a variation of 26.69%, **CHEMBL-263035** (Fig 9B) complex showed a variation of 31.35% in PC1 (Fig 9C). The PC1 in **PubChem-74936833** and **PubChem-44208924** complexes showed variations of 31.22% and 26%, respectively. The PCA analysis, using basic clustering in PC subspace, revealed conformational changes in all clusters; the most significant movement was shown by blue regions, intermediate movement by white regions, and less flexibility by red regions.

#### 3.5.7. MMGBSA

For both complexes, the total binding free energy (ΔG_total_) was estimated by using the empirical equations of the MM/GBSA method. To determine the stability of a protein-ligand complex, the ΔG_total_ energy was calculated [49]. A complex with lower ΔG_total_ values is more stable. It was calculated as the difference between the complex, receptor, and ligand free energies. The combined effect of different protein-ligand interactions, including van der Waals (**ΔE**_**vdW**_), electrostatic energy (**ΔE**_**ele**_), and Generalized Born solvation free energy (**ΔG**_**GB**_**)**, results in the total binding free energy estimated using the MM/GBSA model. The ΔE_vdW_ contribution of the **CHEMBL-263035** complex was higher than that of other complexes, while the electrostatic contribution of the **PubChem-25230613** complex was higher. Similarly, the GB contribution showed that **PubChem-25230613** has a higher GB value than other complexes (Table 6). Interestingly, the total binding free energies hits were more than the control indicating that the hits made a more stable complex than the control.

**Table 6:** The MMGBSA calculations of the complexes.

Energy components	Control	PubChem-25230613	CHEMBL-263035	PubChem-74936833	PubChem-44208924
**ΔE**_**vdW**_	-31.34±0.34	-88.81±0.46	-96.54±0.41	-65.72±0.49	-76.21±0.40
**ΔE**_**ele**_	-4.13±0.33	-45.31±0.57	-41.59±0.55	-30.06±0.39	-32.63±0.51
**ΔE**_**GB**_	19.24±0.44	77.31±0.55	74.12±0.51	54.65±0.43	58.28±0.61
**ΔE**_**surf**_	-3.88±0.04	-9.27±0.04	-10.67±0.03	-7.7±0.05	-8.88±0.04
**ΔG**_**gas**_	-35.47±0.56	-134.12±0.70	-138.13±0.72	-95.78±0.66	-108.85±0.66
**ΔG**_**solv**_	15.35±0.41	68.04±0.52	63.45±0.58	46.95±0.40	49.39±0.59
**TΔS**	-17.89±1.10	-35.82±2.96	-27.43±1.52	-27.32±2.23	-28.86±1.14
**ΔG**_**total**_	-20.12±0.25	-66.08±0.40	-74.68±0.39	-48.83±0.44	-59.45±0.40

#### 3.5.8. Energy decomposition

**Fig 10:**
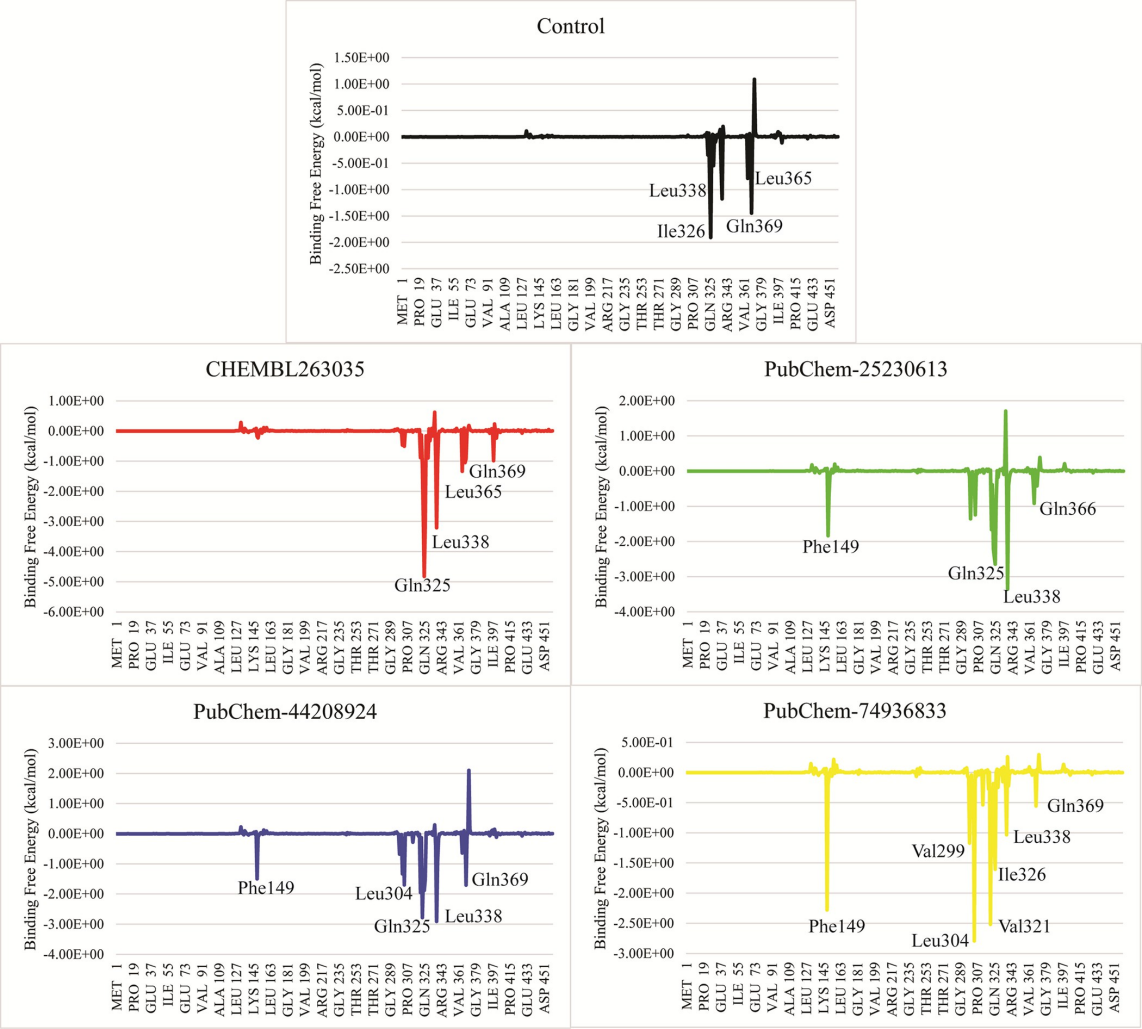
The residual binding free energy decomposition analysis for finding the key interacting residues in all complexes.

To find the contribution of the amino acid residues in total binding free energy, the residue energy decomposition analysis was conducted. The key interacting residues with the control were Ile326, Leu338, Leu365, and Gln369 which contributed to the total binding free energy better than other residues. Similarly, **CHEMBL-263035** showed better interactions with Gln325, Leu338, Leu365, and Gln369. In the case of **PubChem-25230613,** Phe149, Gln325, Leu338, and Gln366 showed better energy contributions. In **PubChem-44208924** complex, the better energy contribution was observed in the residues Phe149, Leu304, Gln325, Leu338, and Gln369. Lastly, Phe149, Val299, Leu304, Val321, Ile326, Leu338, and Gln369 were key interacting residues in the **PubChem-74936833** complex. Upon analysis, it was observed that Gln325 and Leu388 were the key interacting residues in all complexes (Fig 10).

### 3.6 ADMET analysis and drug likeness

The ADMET properties were calculated by QikProp tool. The ADMET terms QPlogPo/w, QPlogHERG, QPPCaco, QPlogBB, and QPlogKhsa were predicted. QPlogPo/w determines the octanol/water partition coefficient, a value within a range of –2.0 to 6.5 is good. The QPlogHERG descriptor refers to the predicted IC50 value for the blockage of HERG K+ channels, which is an indicator for cardiotoxicity [50]. QPPCaco predicts apparent Caco-2 cell permeability in nanometers per second. QPlogBB represents the predicted brain/blood partition coefficient, which is crucial in assessing a compound’s ability to cross the blood-brain barrier and QPlogKhsa refers to the prediction of binding to human serum albumin, with a recommended range of -1.5 to 1.5 for 95% of known drugs. Additionally, the drug-likeness was predicted by midruglikeness which predicted the *in vivo* ability of the compounds to function as drug. *In vivo* probability of compounds shows the likelihood of a compound acting like a successful drug during *in vivo* testing. The predicted values are shown in Table 7.

**Table 7:** The predicted ADMET properties and drug likeness of the control and selected compounds.

	ADMET					Drug-likeness
Compound	QPlogPo/w	QPlogHERG	QPPCaco	QPlogBB	QPlogKhsa	*In vivo* (p)
Control	-1.95	-0.99	1.25	-1.36	-0.86	0.988
CHEMBL263035	2.30	-6.18	30.04	-3.26	-1.12	0.9981
PubChem-25230613	2.72	-7.29	41.10	-2.75	-1.35	0.9982
PubChem-44208924	0.62	-5.28	29.12	-4.63	-1.33	0.9969
PubChem-74936833	-1.07	-6.24	20.05	-4.21	-1.76	0.9762

"QPlogHERG" (<-5), "QPlogPo/w" (-2.0 to 6.5), "QPlogBB" (-3.0 to 1.2), "QPPCaco" (<25 poor, >500 great), and "QPlogKhsa" (-1.5 to 1.5).

## 4. Discussion

*C*. *koseri* infections include urinary tract infections, bloodstream infections, and infections in neonates and immunocompromised people. Prompt and efficient treatment is essential in preventing complications linked to *C*. *koseri* infections. These complications can include organ damage, sepsis, and long-term neurological issues in the case of meningitis. An effective drug can help prevent these complications and improve patient outcomes. Developing effective inhibitors of ATP synthase can provide therapy options for these infections, which can be difficult to control otherwise. Hence, this study was designed to identify novel inhibitors against *C*. *koseri* ATP synthase [13].

The 3D structure of *C*. *koseri* ATP synthase was predicted using SWISS-MODEL, and an ab initio model was also derived via AlphaFold. The predicted protein models’ preliminary structural evaluation was performed. Based on multiple assessment scores, the SWISS-MODEL structure outperformed the AlphaFold model and was selected for pharmacophore screening.

The study of pharmacophores is crucial in drug design because it allows for a more rational and targeted approach to drug development. It aids in the selection, optimization, and development of drug candidates that have a better likelihood of interacting with specific molecular targets, resulting in the development of more effective and safer drugs. Before developing a drug, it is necessary to identify the specific molecular target associated with a disease or condition. Pharmacophore analysis assists in finding the critical properties that a drug must possess to interact effectively with its target [51–54]. The pharmacophore query model was developed using Ampicillin’s pharmacophoric properties involved in molecular interactions with ATP synthase protein. Four pharmacophoric features of ampicillin i.e., hydrogen bond acceptor, hydrogen bond donor, aromatic, and hydrophobic were used to generate the model for virtual screening.

Based on these features, a ligand-based virtual screening of nine databases (ZINC, PubChem, Moleprot, LabNetwork, MCULE-Ultimate, Chemspace, MCULE, ChemDiv, and CHEMBL) was performed, and the hits that met the screening requirements were selected. Ligand-based virtual screening is a computational approach to drug discovery that discovers potential drug candidates based on the chemical and structural properties of known ligands with a specific therapeutic target. This method assumes that compounds with similar structural or chemical features to known ligands would have similar biological actions. When there is limited information about the target structure or acquiring a high-resolution structure of the target protein is challenging, ligand-based virtual screening is very useful. It makes use of the "ligand similarity" concept to find new compounds that may interact with the target [55–58]. A total of 2043 hits were collected from the nine databases combined. The PubChem database generated the greatest number of hits.

The hit compounds found during virtual screening were docked to the ATP synthase receptor to predict binding affinities using the glide docking tool. Molecular docking is used in molecular biology and drug development to forecast and examine the interactions between a target macromolecule (receptor), and a ligand. The main goal of molecular docking is to predict the binding affinity of a ligand. This insight is crucial for understanding how potential drug molecules interact with their target proteins, which can impact the development of new therapies as well as the optimization of existing ones [59–61]. The top ten hits were selected for the molecular interaction analysis based on their docking scores. The chosen compounds had binding affinities in the range of -10.021 to -8.452 kcal/mol. The binding affinities of the selected chemicals suggested that they could impede the function of ATP synthase.

The potential binding mechanisms of the selected compounds were investigated by aligning them with co-crystal ligands. The study revealed that the docked molecules were properly aligned on the co-crystal ligand with a similar binding mechanism. Thus, the feasible binding mechanisms of the selected compounds were subjected to stability analysis using the MD Simulation study. Protein-ligand interactions can be better understood by using MD simulations, which are an effective tool [62]. MD simulations demonstrated the stability of these compounds as potent inhibitors within the protein binding pocket.

The ADMET properties of the identified compounds were predicted using the QikProp tool. This examination provides details about a candidate’s subcellular location, intestinal absorption, blood-brain barrier crossing ability, metabolism, and—above all—the possibility of affecting the body [63–65]. The predicted values indicated that the identified compounds revealed borderline values for QPPCaco and QPlogKhsa. QPPCaco predicts Caco-2 cell permeability, which is a crucial indicator of intestinal drug absorption [66]. The borderline values of the identified compounds suggested moderate permeability, which may impact oral bioavailability. While not optimal, these values do not necessarily preclude further development, as many successful drugs have overcome similar challenges through formulation strategies or structural modifications [67]. QPlogKhsa, predicting binding to human serum albumin, influences drug distribution and half-life [68]. The borderline values indicate moderate protein binding, which could affect the pharmacokinetics of the compounds. However, it is important to note that these QikProp predictions are based on computational models and require experimental validation [69]. Moreover, many marketed drugs have less-than-ideal ADMET properties, suggesting that these borderline values should be viewed as areas for potential optimization rather than disqualifying factors [70].

There are some advantages to computational biology methods, but there are also some disadvantages. For instance, the results generated by different tools can vary, so further investigation and validation in wet labs are required before the results can be trusted [48]. Considering our findings about the bioactivity of certain compounds, more study into lead optimization is required.

## 5. Conclusion

In our investigation, we have discovered new synthetic substances that might have an inhibitory effect on the ATP synthase of *C*. *koseri*. Pharmacophores were used to screen nine small molecule databases, and the best binding modes of screened hits were identified by molecular docking to the ATP synthase protein. The top four compounds (PubChem-25230613, PubChem-74936833, CHEMBL263035, PubChem-44208924) with the best ADMET characteristics and binding modes were chosen. The stability of the docked hits with the protein was evaluated by MD simulation, and conformational changes were detected by RMSD, RMSF which indicated that the hits remained stably bound to the ATP synthase binding pocket. These findings provide important insights into the design and optimization of therapeutics for *C*. *koseri* infections, helping to advance antimicrobial drug discovery and combat antibiotic-resistant pathogens. Further experimental validation and optimization of the identified inhibitors will be critical steps toward clinical application.
